# Family influencing factors of college students’ social anxiety: the role and internal mechanism of parental psychological control

**DOI:** 10.3389/fpsyg.2026.1728108

**Published:** 2026-01-30

**Authors:** Yilin Wei, Rong Mei

**Affiliations:** 1 School of Design and Fashion, Zhejiang University of Science and Technology, Hangzhou, China; 2 School of Humanities, Donghua University, Shanghai, China

**Keywords:** college students, parental psychological control, rejection sensitivity, social anxiety, trait mindfulness

## Abstract

**Objective:**

To explore the relationship between parental psychological control and college students’ social anxiety, and to examine the mediating roles of trait mindfulness and rejection sensitivity.

**Methods:**

A cross-sectional design was used, with data collected from March to June 2025. Convenience sampling was employed to select 586 college students from four universities of varying types in Zhejiang Province, China. Participants completed online questionnaires assessing parental psychological control, trait mindfulness, rejection sensitivity, and social anxiety. Data were analyzed with SPSS 26.0 and the PROCESS macro program (Model 6).

**Results:**

Parental psychological control was positively correlated with social anxiety (*r* = 0.498, *p* < 0.001) and rejection sensitivity (*r* = 0.361, *p* < 0.001), but negatively correlated with trait mindfulness (*r* = −0.483, *p* < 0.001). Trait mindfulness was negatively correlated with social anxiety (*r* = −0.341, *p* < 0.001) and rejection sensitivity (*r* = −0.265, *p* < 0.001), while rejection sensitivity was positively correlated with social anxiety (*r* = 0.560, *p* < 0.001). Three significant mediating paths were revealed: trait mindfulness [*b* = 0.039, 95% CI (0.008, 0.071), *p* = 0.015], rejection sensitivity [*b* = 0.132, 95% CI (0.085, 0.181), *p* < 0.001], and their chain mediation [*b* = 0.025, 95% CI (0.006, 0.044), *p* = 0.010]. The total mediating effect (0.196) accounted for 38.8% of the total effect of parental psychological control on social anxiety.

**Conclusion:**

This study identifies several mechanisms linking parental psychological control to college students’ social anxiety and constructs a chain mediation model that integrates trait mindfulness and rejection sensitivity. It provides empirical support for targeted college mental health interventions.

## Introduction

Social anxiety has long been a key focus in youth mental health research (Gao et al., 2024). Young individuals with social anxiety often exhibit impaired interpersonal skills and academic performance, as well as an increased risk of comorbid psychiatric conditions, such as depression and obsessive-compulsive disorder (OCD) (Schry and White, 2013; Gong et al., 2019; Strahan, 2003).

Research has consistently shown that the family system plays a crucial role in the development of social anxiety (Fu et al., 2025; Zhang et al., 2023), suggesting that familial influences are foundational to understanding this condition. Among various family factors, parenting style is a key influence on psychological development and is strongly associated with social anxiety (Dong et al., 2024). Parental psychological control (PPC) is a form of negative parenting that involves behaviors invading children’s inner world and restricting their autonomy through tactics like guilt induction, withdrawal of love, or authoritarian interference (Barber, 1996; Scharf and Goldner, 2018). Research consistently shows a strong positive correlation between PPC and social anxiety, with higher PPC levels linked to more severe anxiety (Loukas et al., 2005; Falcone et al., 2025).

Why does PPC correlate with social anxiety? What might explain the observed relationship between PPC and social anxiety? Existing research primarily focuses on the direct relationship between PPC and social anxiety (Gao et al., 2022; Rogers et al., 2020), but the underlying mechanisms connecting these two factors have not been thoroughly explored. This study aims to address this research gap by investigating the potential association mechanisms between PPC and social anxiety among college students.

Trait mindfulness and rejection sensitivity correspond to two complementary psychological dimensions: emotional regulation and interpersonal perception. By incorporating both into a unified framework, the multiple pathways underlying the association between PPC and social anxiety in college students can be systematically explained. Trait mindfulness is a stable personality trait that refers to an individual’s ability to perceive and accept present experiences in a non-judgmental manner (Kabat-Zinn, 1994). Research has shown a negative correlation between PPC and trait mindfulness, with higher levels of PPC being associated with lower levels of trait mindfulness. This is because psychological control behaviors hinder the autonomous awareness needed for mindfulness, thereby impeding its development (Li et al., 2024).

Additionally, research has shown that rejection sensitivity is a significant factor associated with increased social anxiety. Rejection sensitivity refers to an individual’s heightened vigilance, anticipation, and emotional reactions to potential rejection cues in social interactions (Downey and Feldman, 1996). Individuals with high rejection sensitivity often experience strong negative expectations and anxiety due to persistent fears of rejection (Zimmer-Gembeck et al., 2021).

Based on the aforementioned research, it can be inferred that trait mindfulness and rejection sensitivity may play significant roles in the relationship between PPC and social anxiety among college students. Furthermore, studies have shown a significant association between trait mindfulness and rejection sensitivity (Ghonchehpour et al., 2023). By enhancing present-moment awareness and emotion regulation, trait mindfulness may be related to reduced vigilance toward negative social cues, which could be associated with lower rejection sensitivity. Based on these findings, it is reasonable to hypothesize that correlations exist among PPC, trait mindfulness, rejection sensitivity, and social anxiety. Specifically, higher levels of PPC are associated with lower levels of trait mindfulness, which is also associated with greater rejection sensitivity and higher levels of social anxiety. While existing studies have explored the relationships between PPC and both trait mindfulness and rejection sensitivity, they have not clarified the specific mechanisms linking these variables to social anxiety in college students.

Based on this, the present study constructs a parallel and serial mediation model to examine the roles of trait mindfulness and rejection sensitivity in the relationship between PPC and social anxiety among college students. The following sections will further elaborate on the theoretical basis of this study and provide a more in-depth explanation of the research variables.

## Theoretical framework and hypothesis

Parental psychological control, characterized by intrusive parenting, has long been linked to negative psychological outcomes in emerging adults (Barber, 1996). According to ecological systems theory (Bronfenbrenner, 1979), PPC tends to coincide with limited autonomy support within the family microsystem. Individuals exposed to such parenting practices are more likely to develop cognitive biases, such as contingent self-worth based on others’ approval. In social contexts, this often leads to excessive attention to negative feedback, resulting in higher levels of social anxiety (Nanda et al., 2012).

Further empirical studies have confirmed the direct association between PPC and social anxiety. A four-wave longitudinal study of 4,731 Chinese adolescents (Zhou et al., 2024) found that PPC positively predicts social anxiety levels, with a mutually reinforcing cycle between the two variables. Similarly, a cross-sectional study of 1,343 Chinese middle school students (Fu et al., 2025) found a significant positive correlation between PPC and social anxiety.

The underlying mechanism of this association can be summarized in two key aspects. First, individuals exposed to high levels of parental emotional manipulation are more likely to develop a conditional acceptance mindset, which heightens their fear of disapproval in social interactions. Second, prolonged deprivation of autonomy is often associated with poor social decision-making skills, making individuals more susceptible to anxiety arising from uncertainty about how to respond in social situations.

Based on the foregoing analysis, this study proposes the following hypothesis:

*Hypothesis 1*: PPC is significantly and positively correlated with social anxiety levels among college students.

Trait mindfulness may be negatively correlated with social anxiety among college students. As a stable personality trait, trait mindfulness is characterized by non-judgmental and accepting awareness of one’s present-moment physical and mental experiences (thoughts, feelings, bodily sensations) (Brown and Ryan, 2003). Its core dimensions, particularly experience description and acceptance, are significantly negatively associated with social anxiety schemas (Parsons et al., 2017). Drawing on the theoretical framework of Mindfulness-Based Stress Reduction, individuals with high trait mindfulness are less likely to ruminate on past negative experiences or worry about future outcomes. Specifically, they are better at interpreting ambiguous social cues objectively, without equating them with threats to self-worth (Reibel and McCown, 2019; Brown and Ryan, 2003). Furthermore, empirical studies have shown that the negative correlation between trait mindfulness and anxiety is not significantly confounded by the Big Five personality traits or cognitive abilities (Banfi and Randall, 2022). These findings suggest that the anxiety-reducing effect of trait mindfulness is stable and independent.

PPC may impair individuals’ present-moment experiences, reducing their levels of trait mindfulness (Kabat-Zinn, 2023). According to Self-Determination Theory, mindfulness development requires an autonomously supportive family environment. Parental respect for children’s thoughts and emotions helps them develop the habit of attending to their inner experiences, fostering acute awareness of emotions, cognitions, and bodily sensations. This, in turn, lays the foundation for the stable development of trait mindfulness (Deci and Ryan, 2000). In contrast, PPC imposes external evaluative pressure, pushing individuals to prioritize fulfilling parental expectations over attending to their authentic feelings (Li et al., 2024). Over time, this pressure diminishes their capacity for present-moment awareness. Individuals with low trait mindfulness are more likely to neglect their internal experiences when exposed to parental emotional manipulation or autonomy deprivation, falling into a vicious cycle of failing to recognize emotional needs and struggling to regulate psychological stress (Han et al., 2021). This cycle further reinforces low mindfulness levels, creating a negative feedback loop.

Based on the above analysis, it is reasonable to infer that trait mindfulness may mediate the relationship between PPC and social anxiety among college students. Therefore, the present study proposes the following hypothesis:

*Hypothesis 2*: Trait mindfulness mediates the relationship between PPC and social anxiety among college students.

Parental psychological control is likely positively correlated with rejection sensitivity. Rejection sensitivity refers to a dispositional tendency characterized by heightened vigilance toward potential rejection cues. This sensitivity is believed to stem from conditional parental acceptance during early childhood (Downey and Feldman, 1996). According to attachment theory, early parent-child interactions shape individuals’ interpersonal expectations and relational schemas. Childhood experiences with parents significantly influence cognitive appraisals and anticipations, which in turn affect later interpersonal relationships (Zeanah, 1990).

Parental psychological control typically manifests through behaviors such as guilt induction and withdrawal of love. Its core feature is the transmission of conditional acceptance signals—children must meet specific demands to gain emotional approval. This pattern fosters the expectation that acceptance depends on personal performance. Compared to children who receive unconditional acceptance, these individuals experience greater uncertainty regarding interpersonal acceptance, making them more sensitive to rejection cues.

From a cognitive development perspective, these interpersonal expectations solidify into two stable cognitive predispositions. First, they shape negative interpersonal cognitive schemas and increase vigilance toward rejection signals (Jain and Sharma, 2025). Second, they lead to the core belief that “if I am not good enough, I will be rejected,” prompting individuals to excessively seek rejection cues during social interactions (Lian et al., 2021).

As a stable interpersonal cognitive predisposition, rejection sensitivity is closely linked to social anxiety in college students. Specifically, it influences social anxiety through two pathways: negative social risk perception and the self-fulfilling prophecy of social avoidance (Downey et al., 2004). On the one hand, individuals with high rejection sensitivity may overestimate the likelihood of rejection in social situations, leading to feelings of insecurity in relationships and potentially increasing social anxiety (Fang et al., 2011). On the other hand, rejection sensitivity may contribute to the self-fulfilling prophecy of social avoidance. Fear of rejection can cause individuals to avoid social situations, leading to a lack of social experience, which reinforces their belief that “I will be rejected” and exacerbates social anxiety. A five-year longitudinal study has confirmed that rejection sensitivity can independently predict the development of social anxiety symptoms in adolescents (Zimmer-Gembeck et al., 2021).

In summary, PPC may be associated with rejection sensitivity through conditional acceptance and the shaping of negative cognitive patterns. Rejection sensitivity, in turn, may contribute to social anxiety. Therefore, rejection sensitivity may mediate the relationship between PPC and social anxiety in college students. Based on this, we propose the following hypothesis:

*Hypothesis 3*: Rejection sensitivity mediates the relationship between PPC and social anxiety among college students.

Trait mindfulness may be negatively correlated with rejection sensitivity and form a chain mediation model with PPC and social anxiety. According to Attention Resource Allocation Theory, trait mindfulness is a fundamental cognitive trait characterized by decentering and present-moment awareness (Brown et al., 2007), constituting deep-seated cognitive regulation resources. In contrast, rejection sensitivity is a surface-level cognitive bias developed through repeated interpersonal interactions. Basic cognitive resources shape surface-level interpersonal cognitive tendencies. Thus, trait mindfulness plays a positive regulatory role in the formation and externalization of rejection sensitivity.

Additionally, according to the Conservation of Resources Theory (Hobfoll, 1989) in developmental psychology, early parenting practices, such as PPC, play a crucial role in shaping core psychological resources like trait mindfulness in individuals. Subsequently, based on the available resources of the individual, these resources become associated with specific interpersonal perceptual traits, such as rejection sensitivity. This developmental trajectory validates the sequential relationships between the variables in the proposed chain mediation model.

The theoretical perspective of Mindfulness-Based Cognitive Therapy further elaborates on the mechanism underlying this mediating chain. Individuals with high trait mindfulness possess stronger decentering abilities, enabling them to effectively decouple others’ behaviors from their self-worth, thereby avoiding the interpretation of ambiguous social cues as rejection signals (Segal et al., 2012). Conversely, individuals with low trait mindfulness exhibit a higher degree of self-involvement, making them more inclined to over-associate others’ behaviors with their self-evaluation, which correlates with higher rejection sensitivity.

This mechanism is supported by intervention studies. A mindfulness intervention with 16 adolescents who had experienced childhood trauma showed significant improvement in mindfulness and a decrease in rejection sensitivity after the intervention. Notably, improvement in non-attachment—a core dimension of mindfulness—was a key factor driving this effect (Joss et al., 2020). Another randomized controlled trial involving 66 thalassemia patients confirmed that mindfulness interventions effectively reduce rejection sensitivity across diverse populations (Ghonchehpour et al., 2023).

Based on the above evidence, it can be inferred that trait mindfulness and rejection sensitivity mediate the relationship between PPC and social anxiety in college students. Specifically, PPC is associated with trait mindfulness and, through its negative association with trait mindfulness, with increased rejection sensitivity. In turn, this heightened rejection sensitivity correlates with elevated levels of social anxiety. Therefore, the present study proposes the following hypothesis:

*Hypothesis 4*: Trait mindfulness and rejection sensitivity mediate the relationship between PPC and social anxiety among college students.

**Figure 1:**
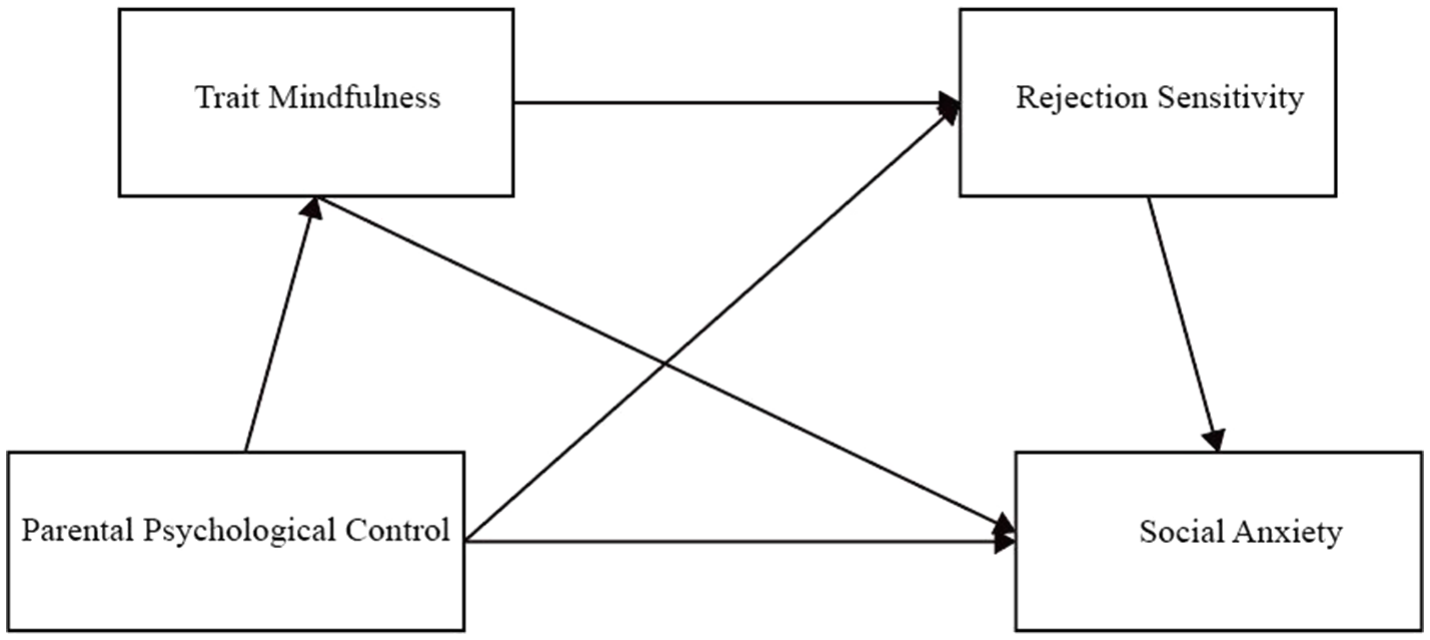
Hypothetical model.

The hypothetical model of this study is shown in Figure 1.

## Methods

### Research design

This study used a cross-sectional survey design to examine the relationships among PPC, trait mindfulness, rejection sensitivity, and social anxiety in college students. Data were collected via an online questionnaire survey. The proposed chain mediation model was tested using structural equation modeling (SEM) along with the PROCESS macro program.

### Participants

Considering resource accessibility and sample diversity, this study used a convenience sampling method to select four representative universities in Zhejiang Province. These included comprehensive universities, science and engineering schools, liberal arts colleges, and art institutions. This approach aimed to reduce sampling bias by ensuring a diverse sample from different types of institutions. This study did not use probability sampling or stratified sampling for the following reasons: (1) the complete student enrollment lists required for probability sampling could not be obtained due to ethical and authorization restrictions. (2) cross-institutional stratified sampling involves high operational costs and non-uniform standards, making it difficult to complete data collection within a short time frame.

Questionnaires were distributed via the online platform “Wenjuanxing” from March to June 2025. A total of 680 questionnaires were distributed, 633 were returned, and 586 were valid, yielding an effective response rate of 86.18% and an overall recovery rate of 93.09%. The valid sample included a diverse group of students in terms of gender, grade, major, hometown, and household income (see Table 1 for details).

**Table 1:** Demographic characteristics of the valid samples (*n* = 586).

Demographic variable	Category	*n*	Percentage (%)
Gender	Male	269	45.90
	Female	317	54.10
Grade	Freshman	175	29.86
	Sophomore	167	28.50
	Junior	149	25.43
	Senior	95	16.21
Major field	Liberal arts	212	36.18
	Science	188	32.08
	Engineering	127	21.50
	Art	59	10.07
Hometown	Urban	326	55.60
	Rural	260	44.40
Monthly household income (RMB)	≤8,000	205	35.00
	8,001–15,000	283	48.30
	≥15,001	98	16.70

The inclusion criteria were: (1) participants were undergraduate students (freshman to senior) enrolled at the selected universities; (2) participants voluntarily agreed to participate in the study; (3) participants were able to complete the questionnaire independently. The exclusion criteria were: (1) incomplete questionnaires with three or more missing responses; (2) missing data on key variables; (3) obvious response biases, such as straight-lining or extreme response patterns; (4) failure to provide informed consent.

### Sample size

An *a priori* power analysis using G*Power 3.1 indicated that the required minimum sample size was 427, based on a medium effect size (*f*^2^ = 0.15), *α* = 0.05, and power (1 − *β*) = 0.95. The final effective sample size was 586, which exceeded the minimum requirement and was sufficient for subsequent analyses.

### Ethical approval

This study was approved by the Ethics Committee of Zhejiang University of Science and Technology. Participants were provided with electronic informed consent, with the assurance that they could withdraw at any time and that their data would be anonymized. Only after participants voluntarily signed the informed consent forms were they allowed to proceed to the questionnaire section.

### Quality control

In terms of quality control, this study ensured the applicability of the scales through pre-tests and validation. A pre-test with 60 participants confirmed the applicability of the Social Anxiety Scale for Adolescents to college students, yielding an average applicability score of ≥ 4 (on a 5-point scale) and a Cronbach’s *α* of 0.87. Confirmatory factor analysis (CFA) was conducted on the Social Anxiety Scale data from the main sample, showing a good model fit: *χ*^2^/d*f* = 1.649 (<3), GFI = 0.973 (>0.90), RMSEA = 0.033 (<0.08), RMR = 0.020 (<0.05), CFI = 0.995 (>0.90), and NFI = 0.988 (>0.90). The factor loadings for all items were significantly greater than 0.8, indicating excellent discriminability. Convergent validity was good, with an average variance extracted (AVE) = 0.752 (>0.5) and composite reliability (CR) = 0.975 (>0.7). Furthermore, the square roots of the AVE for each factor were greater than the inter-factor correlation coefficients, confirming discriminant validity. In summary, these results suggest that the Social Anxiety Scale for Adolescents is suitable for the college student sample in this study.

The other three scales (PPC Questionnaire, Mindful Attention Awareness Scale, and Rejection Sensitivity Scale) were not pre-tested, as their applicability, reliability, and validity have already been confirmed in several studies conducted with Chinese college student samples.

### Research instruments

#### Parental psychological control questionnaire

Parental psychological control was measured using the Children’s Report of Parental Behavior Inventory (CRPBI), originally developed by Schaefer (1965) and later revised by Wang et al. (2007) for the Chinese population, which was adopted in this study. The questionnaire assesses PPC across three dimensions: guilt induction (e.g., “When I fail to meet my parents’ requirements, they make me feel guilty”), love withdrawal (e.g., “When I do not obey them, my parents reduce their care for me”), and authoritarian power (e.g., “My parents require me to do everything exactly as they think”). The questionnaire consists of 18 items, rated on a 5-point Likert scale (1 = “completely inconsistent” to 5 = “completely consistent”), with all items scored positively. A higher total score reflects a higher level of perceived PPC. This version of the questionnaire has been validated for good reliability and validity and is widely used in adolescent populations, thus it was adopted in this study. The Cronbach’s *α* coefficient for this questionnaire in this study was 0.93.

#### Mindful attention awareness scale

Mindfulness was measured using the Mindful Attention Awareness Scale (MAAS), developed by Brown and Ryan (2003), with the Chinese version revised by Chen et al. (2012) employed in this study. The scale has a unidimensional structure consisting of 15 items, which focus on “non-judgmental awareness of present-moment experiences.” Example items include reverse-scored items (e.g., “I often get distracted when doing things and cannot notice my current feelings”) and positively scored items (e.g., “I can be aware of changes in my current emotions”). All items were rated on a 6-point Likert scale (1 = “almost always” to 6 = “almost never”). To control for potential method effects caused by reverse-scored items, CFA was conducted to compare the fit of two models: one including a common method factor related to reverse-scored items and the other excluding this factor. The results indicated that adding the method factor only slightly improved the model fit (ΔCFI = 0.002, ΔRMSEA = 0.001), and the factor loadings of all items remained stable (0.69–0.82), indicating that reverse-scored items did not introduce substantial method bias. The total score of the scale was calculated by summing the scores of all items (including reverse-coded items), with a higher total score reflecting a higher level of trait mindfulness. The Cronbach’s *α* coefficient for this scale in this study was 0.88.

#### Rejection sensitivity scale

Rejection sensitivity was measured using the Rejection Sensitivity Scale developed by Jobe (2003) and the Chinese version revised by Hu (2017), which was employed in this study. The questionnaire consists of 18 items, rated on a 5-point Likert scale (1 = “completely inconsistent” to 5 = “completely consistent”), covering three dimensions: emotional response (e.g., “When rejected by others, I feel extremely hurt”), interpersonal confidence (e.g., “I am not sure whether I can be accepted by others”), and need for acceptance (e.g., “I strongly hope to gain recognition from people around me”). A higher total score reflects stronger rejection sensitivity. The Cronbach’s *α* coefficient for this scale in this study was 0.83.

#### Social anxiety scale for adolescents

Social anxiety was measured using the Social Anxiety Scale for Adolescents (SAS-A) developed by La Greca (1999), with the Chinese version translated and revised by Zhu (2008) employed in this study. There are two main reasons for choosing this scale: first, its three dimensions—fear of negative evaluation, social avoidance and distress—accurately reflect the core sources of social anxiety in college students, in line with their developmental characteristics during the transition from adolescence to adulthood; second, its concise items reduce respondent fatigue and focus on subclinical anxiety assessment, which aligns with the objectives of this study. In contrast, other alternative scales (e.g., LSAS) focus on clinical or occupational settings, while unidimensional scales such as SIAS and SPS have limited applicability to campus settings. The scale includes 13 items across the three dimensions mentioned above. Representative items include fear of negative evaluation (e.g., “I worry that others will think I am doing a bad job”), social avoidance and distress with strangers (e.g., “I feel nervous when communicating with people I do not know”), and social avoidance and distress with acquaintances (e.g., “Even when I am with people I know very well, I often feel uncomfortable”). All items were rated on a 5-point Likert scale (1 = “completely inconsistent” to 5 = “completely consistent”) and scored positively. The total score is the sum of the scores from the three dimensions, with a higher total score reflecting a higher level of social anxiety. The Cronbach’s *α* coefficient for this scale in this study was 0.89.

### Data analysis

Data analysis for this study was conducted using SPSS 26.0, AMOS 26.0, and the PROCESS macro program (Hayes, 2013), following these steps: First, descriptive statistics, reliability analysis, and Pearson correlation analysis were performed in SPSS 26.0 to examine the distribution and relationships among variables. Second, a SEM was constructed in AMOS 26.0, and CFA was conducted to validate the measurement model, assessing the reliability, validity, and overall fit of the latent variables. This provided the foundation for the subsequent mediation tests. The PROCESS macro (Model 6) was then used to test the chain mediating effect of trait mindfulness and rejection sensitivity between PPC and social anxiety in college students. The reason for choosing the PROCESS macro and Model 6 is that it provides bias-corrected bootstrap estimates of indirect effects, which is suitable for testing complex mediation models. Additionally, Model 6 is specifically designed for chain mediation models, making it well-suited for testing the research hypothesis in this study. Finally, the Bootstrap method with 5,000 resamples was used to calculate the 95% confidence interval (CI), with a mediating effect considered significant if the CI did not include 0.

## Results

### Preliminary analysis

Harman’s single-factor test was conducted to assess the impact of common method bias. The results revealed that four common factors with eigenvalues greater than 1 were extracted, with the first common factor accounting for 19.993% of the variance. Therefore, no significant common method variance was found in this study.

Table 2 presents the means, standard deviations, and Pearson correlation coefficients for all study variables. Significant positive correlations were found between PPC and both social anxiety and rejection sensitivity, while PPC showed a significant negative correlation with trait mindfulness. Trait mindfulness was negatively correlated with both social anxiety and rejection sensitivity, and rejection sensitivity was positively correlated with social anxiety.

**Table 2:** Pearson correlation, CR, and AVE.

Variables	*M*	SD	AVE	CR	1	2	3	4
1. (PPC)	2.523	1.157	0.691	0.976	**0.831**			
2. (Trait mindfulness)	4.530	1.301	0.739	0.977	−0.483^**^	**0.860**		
3. (Rejection sensitivity)	4.182	0.846	0.643	0.970	0.361^**^	−0.265^**^	**0.802**	
4. (Social anxiety)	3.896	1.174	0.752	0.975	0.498^**^	−0.341^**^	0.560^**^	**0.867**

^*^p < 0.05, ^**^p < 0.01. The bold numbers are the square root values of AVE.

### Validation of the measurement model

Confirmatory factor analysis was conducted on the measurement model using AMOS 26.0, which included four latent variables: PPC, trait mindfulness, rejection sensitivity, and social anxiety. The model showed good fit indices: *χ*^2^ = 2799.698, df = 1946, *χ*^2^/df = 1.439, *p* < 0.001, RMSEA (90% CI) = 0.027 (0.025–0.030), SRMR = 0.026, CFI = 0.878, TLI = 0.977.

Although the CFI value of 0.878 was slightly below the conventional cutoff of 0.90, this can be attributed to the model’s complexity, which includes four latent variables and numerous observed items (df = 1946). However, all core fit indices met the relevant criteria: the *χ*^2^/df ratio of 1.439 was within the acceptable range; RMSEA = 0.027 [90% CI (0.025, 0.030)] and SRMR = 0.026 were well below their thresholds; and the TLI value of 0.977 confirmed the model’s relative fit quality.

To test the cross-group stability of the measurement model, gender and grade were used as grouping variables to examine configural, metric, and scalar invariance, using the criteria ΔCFI ≤ 0.01 and ΔRMSEA ≤ 0.015. Results showed that invariance tests at all levels for both gender and grade groupings met the specified criteria (ΔCFI = 0.003–0.006, ΔRMSEA = 0.001–0.002). This indicates that the measurement model exhibited consistent properties across groups, supporting cross-group comparisons of structural paths.

All factor loadings of the observed variables were significant (*p*s < 0.001, range: 0.69–0.88). For convergent validity, the CR of the latent variables ranged from 0.970 to 0.977 (all >0.70), and the AVE ranged from 0.643 to 0.752 (all >0.50). For discriminant validity, the square root of the AVE for each latent variable was greater than its correlation with other latent variables (see Table 2), confirming the scale’s good reliability and validity.

### Test of mediating effects

Mediating effects were assessed using the bias-corrected percentile Bootstrap method with 5,000 resamples and a 95% CI, as implemented in the PROCESS macro (Model 6). The results (Table 3) indicated that all three mediating paths were statistically significant, as none of their 95% CIs included zero.

**Table 3:** Summary of effect analysis process.

Effect	Effect	SE	*t*	*p*	LLCI	ULCI
Direct effect	0.309	0.038	8.124	0.000	0.235	0.384
Total effect	0.505	0.036	13.883	0.000	0.434	0.577
Indirect effect 1	0.039	0.016	2.421	0.015	0.008	0.071
Indirect effect 2	0.132	0.025	5.365	0.000	0.085	0.181
Indirect effect 3	0.025	0.010	2.574	0.010	0.006	0.044
Total indirect effect	0.196	0.027	7.332	0.000	0.142	0.244

The path of indirect effect 1 is PPC-trait mindfulness-social anxiety; the path of indirect effect 2 is PPC-rejection sensitivity-social anxiety; and the path of indirect effect 3 is PPC-trait mindfulness-rejection sensitivity-social anxiety.

The parallel mediation model revealed two distinct pathways: (1) the trait mindfulness-mediated path runs from PPC to trait mindfulness and then to social anxiety, showing a negative mediating pattern (indirect effect = 0.039). In this path, PPC was negatively associated with trait mindfulness (*β* = −0.543, *p* < 0.001), and trait mindfulness was negatively associated with social anxiety (*β* = −0.072, *p* = 0.028). (2) The rejection sensitivity-mediated path, with the largest effect size, exhibited a positive mediating pattern (indirect effect = 0.132). This path runs from PPC to rejection sensitivity and then to social anxiety. Here, PPC was positively linked to rejection sensitivity (*β* = 0.222, *p* < 0.001), and rejection sensitivity was positively associated with social anxiety (*β* = 0.595, *p* < 0.001).

In addition to the parallel mediating paths, the serial mediating path (from PPC to trait mindfulness, then to rejection sensitivity, and finally to social anxiety) was also significant (indirect effect = 0.025, *p* = 0.010), reflecting a sequential association mechanism. Specifically, higher levels of PPC were associated with lower trait mindfulness (*β* = −0.543, *p* < 0.001), and lower trait mindfulness was associated with higher rejection sensitivity (*β* = −0.077, *p* = 0.007), which in turn, was positively associated with social anxiety (*β* = 0.595, *p* < 0.001).

**Figure 2:**
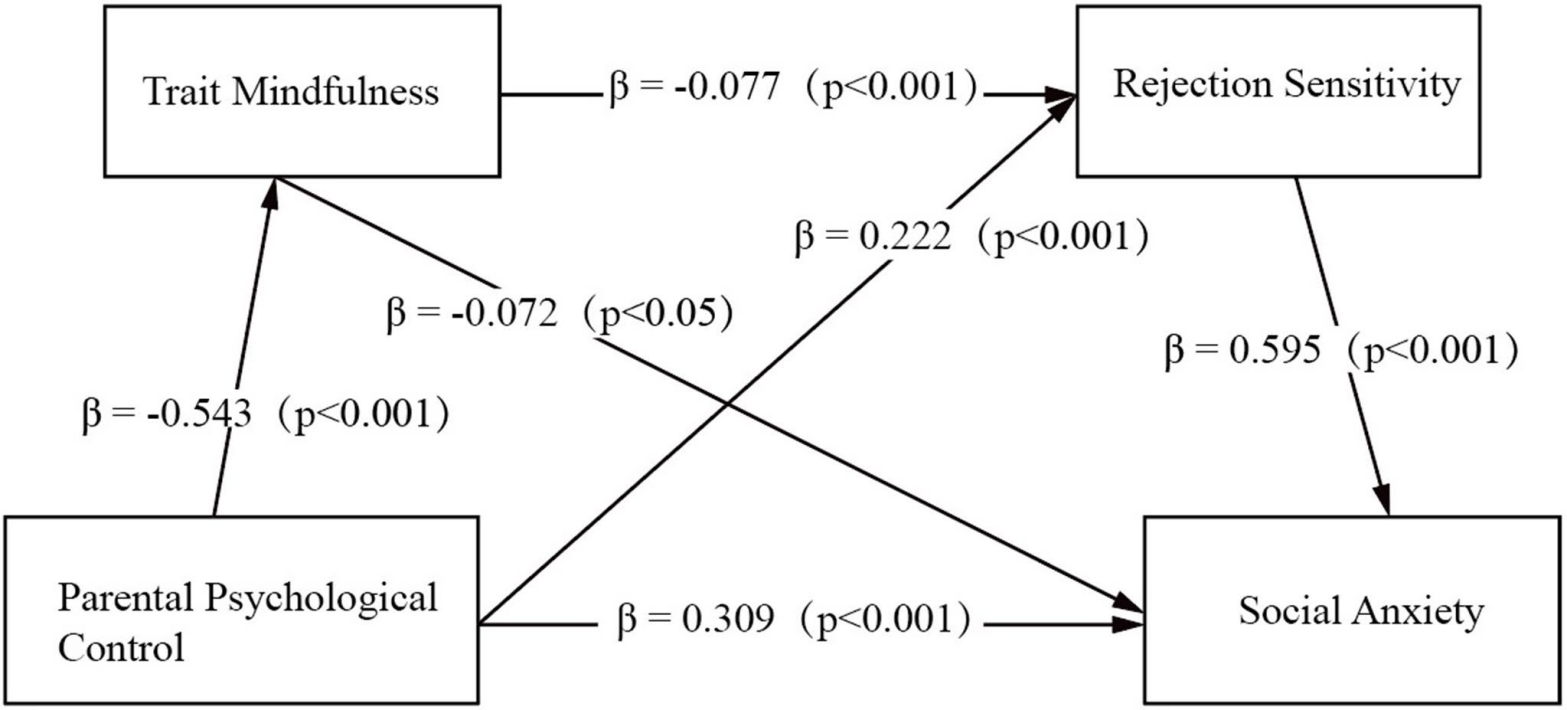
Mediating effect model.

A comparison of the three mediating effects revealed that rejection sensitivity had the largest effect (0.132), followed by trait mindfulness (0.039), and the serial path had the smallest effect (0.025). The total indirect effect (0.196) accounted for 38.8% of the total relationship between PPC and social anxiety (0.505). The direct effect of PPC on social anxiety remained significant (*β* = 0.309, *p* < 0.001). These results confirm that trait mindfulness and rejection sensitivity play partial mediating roles in the relationship between PPC and college students’ social anxiety. The mediating effect model is presented in Figure 2.

## Discussion

### Overview of core findings

Guided by attachment theory and self-determination theory, this study explored the relationship between PPC and social anxiety among college students, with a focus on the mediating roles of trait mindfulness and rejection sensitivity. The results revealed a significant positive correlation between PPC and social anxiety, and confirmed the three hypothesized mediating pathways: (1) PPC was negatively associated with trait mindfulness, which, in turn, was positively related to social anxiety; (2) PPC was positively associated with rejection sensitivity, which, in turn, was positively correlated with social anxiety; (3) PPC was negatively associated with trait mindfulness, which was positively related to both rejection sensitivity and social anxiety.

### Interpretation of main effect and independent mediating effects

The present study found a significant positive correlation between PPC and social anxiety among college students, a finding consistent with previous research (Zhou et al., 2024; Loukas et al., 2005). Compared to peers with lower levels of PPC, individuals in high-control environments are at greater risk of experiencing suppressed autonomy, restricted emotional expression, and excessive parental control over social behavior choices (Barber, 1996). These individuals often lack opportunities to independently explore social situations, struggle to develop flexible interpersonal coping skills, and feel uncertain during social interactions. Additionally, they tend to have difficulty establishing stable social confidence, are more likely to worry about not meeting others’ expectations, and are prone to social anxiety. This result is consistent with attachment theory, which posits that the insecure parent-child relationship created by PPC influences individuals’ perceptions of social risks, thereby contributing to their level of social anxiety (Zeanah, 1990).

This study conducted a cross-group comparison with Zhou et al.’s (2024) research on Chinese adolescents. The results showed that although the correlation coefficients differed in value, both studies indicated a significant positive correlation between PPC and social anxiety, validating the cross-group applicability of this association effect. The discrepancy in effect sizes might stem from two aspects. First, differences in developmental stages could account for the discrepancy. Adolescents are embedded in the core context of family life, so the effect of PPC accumulates gradually through daily interactions. In contrast, although college students live independently away from family, complex campus social contexts (e.g., dormitory relationships, club interactions) might still activate the long-term effects of early PPC. Second, the difference in research design: this study adopted a cross-sectional design, focusing on the strength of variable associations at specific time points; in contrast, Zhou et al. (2024) employed a longitudinal design, emphasizing the exploration of long-term developmental trends of the association. Such differences in research focus might lead to variations in effect sizes. The above speculations regarding the causes of effect size differences await further verification in subsequent studies.

The results of this study confirmed that both trait mindfulness and rejection sensitivity mediate the relationship between PPC and social anxiety, with trait mindfulness representing psychological resource status and rejection sensitivity reflecting interpersonal perception bias. Specifically, the mediating effect of trait mindfulness was observed as follows: PPC was negatively correlated with individuals’ capacity for mindful awareness and experiential acceptance. In contrast, lower levels of mindfulness were positively correlated with cognitive tendencies such as social rumination and excessive worry. These two associations together formed the basis for the accumulation of anxiety (Reibel and McCown, 2019). This finding highlights the role of psychological resources in mediating the relationship between PPC and social anxiety. While previous studies have confirmed the link between parental control and emotion regulation (Fu et al., 2025), this study provides a clearer understanding of the mediating chain among these three variables, offering new insights into the indirect pathway linking parental control and social anxiety.

The mediating effect of rejection sensitivity demonstrated that conditional acceptance from parents tended to lead individuals to form the cognition that “self-worth depends on others’ attitudes”. This belief was positively associated with increased vigilance toward social rejection cues, which in turn contributed to social anxiety through a vicious cycle of social avoidance and reinforcement of negative beliefs (Downey and Feldman, 1996; Noda et al., 2022). This finding highlighted the mediating role of interpersonal perception bias. Previous studies had found an association between PPC and social adaptation problems among first-year college students (Zou et al., 2023), and this study further expanded the research perspective by clarifying the mediating role of rejection sensitivity, confirming that it was a key pathway linking PPC and social anxiety.

Notably, the mediating effect of rejection sensitivity was stronger, with a significant difference in effect sizes between the two mediators (rejection sensitivity: 0.132; trait mindfulness: 0.039). This may be due to the direct and immediate nature of rejection sensitivity, whereas trait mindfulness serves as a more distal psychological resource, yielding a weaker effect in a cross-sectional context.

### In-depth analysis of the chain mediating effect

The results of the chain-mediation analysis supported the hypothesis, revealing the sequential mediating pathway between PPC and social anxiety. Specifically, PPC was negatively associated with trait mindfulness, which in turn was negatively associated with rejection sensitivity. Ultimately, rejection sensitivity was positively associated with social anxiety. The core “awareness-acceptance” feature of trait mindfulness is negatively correlated with bias in interpersonal perception and is also associated with individuals’ tendency to objectively perceive their emotional and cognitive responses, reducing over-interpretation of social cues and decreasing rejection sensitivity.

This pathway strengthens the explanatory power of attachment theory by linking early parenting experiences to psychological resources, highlighting their association with interpersonal perception and adaptation in emerging adulthood. It suggests a developmental mechanism: early parenting experiences are associated with psychological resources, which may be related to interpersonal perception and adaptation. Previous studies have primarily focused on the relationship between early parenting and social adaptation in emerging adulthood. However, there has been limited exploration of how psychological resources influence interpersonal perception biases. This study further investigates the relationship between PPC and social anxiety, particularly the role of trait mindfulness and rejection sensitivity. These analyses provide new insights into the key factors of this transitional phase and deepen the application of attachment theory in emerging adulthood.

This result is consistent with previous studies suggesting that early parenting styles may shape individuals’ interpersonal risk perception patterns through psychological resource reserves, which may also be linked to their emotional adaptation (Segal et al., 2012; Jain and Sharma, 2025). Previous studies have mostly focused on single mediators (Fu et al., 2025; Zou et al., 2023), whereas this study identified multiple pathways linking PPC to social anxiety, thereby clarifying the distinct roles of different mediators. This provides valuable insights for the precise intervention of social anxiety among college students affected by PPC.

### Theoretical contributions

The theoretical contributions of this study are reflected in three aspects. First, it identified the dual and chain mediating pathways of trait mindfulness and rejection sensitivity, applying the theoretical framework of family parenting and social adaptation in emerging adulthood to the campus social context of college students. This finding broadened the scope of application of the theoretical framework and provided empirical support for its use in emerging adulthood. Second, it illuminated the transmission mechanism from trait mindfulness to rejection sensitivity through chain-mediation analysis. This mechanism highlighted the practical implications of mindfulness theory in the development of interpersonal perception bias, providing new empirical evidence for its expansion. Third, it clarified the central role of rejection sensitivity in the chain of associations between early parenting styles, psychological resources, and social anxiety in adulthood. This finding expanded the theoretical applications of rejection sensitivity theory, deepened the understanding of its mechanisms in the context of family parenting and psychological adaptation, and provided a new empirical perspective for integrating related theories.

### Practical contributions

Based on the study’s findings, implications for college mental health education and family parenting guidance can be derived in three ways. First, colleges and universities can offer structured mindfulness training programs, such as 8–10 week courses that include evidence-based interventions like mindfulness meditation and body scan practices. These programs would help students improve their ability to maintain non-judgmental awareness, reducing social rumination and negative expectations, and thus weakening the correlation chain of low mindfulness, high rejection sensitivity, and high anxiety. Second, for college students with a history of parental control, colleges could implement group interventions targeting rejection sensitivity. These interventions could include cognitive restructuring and social skills training to help students reduce their biases in perceiving interpersonal risks. Third, schools could establish a school-family collaboration model for parenting education, promoting the concept of autonomy-supportive parenting. This would aim to reduce psychological control behaviors, such as emotional manipulation and autonomy deprivation.

### Research limitations

The limitations of this study are as follows. First, the cross-sectional research design limited the ability to infer causal relationships among variables. It also could not rule out the possibility of reverse causality, such as social anxiety being negatively correlated with trait mindfulness or rejection sensitivity. Future research could adopt a two-wave longitudinal design with a 6 month interval, using cross-lagged models to examine the temporal dynamics of the chain-mediated pathway. Second, the study used non-probability and non-stratified sampling, which may have resulted in an unbalanced distribution of the sample across dimensions such as grade level, place of origin, and family socioeconomic status. Future studies could use multi-stage stratified random sampling, supported by university administrative data, to enhance the sample’s geographic and demographic diversity, thus improving the generalizability of the findings. Third, variable measurement relied solely on self-report scales, which may be susceptible to common method biases, such as social desirability and response biases. Future research could incorporate multi-source data (e.g., peer evaluations) and behavioral experiments to improve measurement objectivity. Fourth, the analysis did not include potential moderating variables (e.g., social support, coping styles) or explore the specific dimensions of PPC (e.g., emotional manipulation, negative evaluation) in relation to social anxiety. Future studies could use moderated mediation models and conduct more detailed analyses of the dimensional impacts of PPC.

## Conclusion

This study analyzed the mechanisms through which PPC is related to social anxiety in college students using a chain mediation model. The findings indicate that trait mindfulness and rejection sensitivity serve both independently and collectively as mediators in this relationship. These results not only enrich the theoretical understanding of family parenting and social adaptation but also offer practical guidance for college mental health education and family parenting programs. Future research should further validate the model with more rigorous designs to provide a stronger scientific basis for reducing social anxiety and promoting social adaptation among college students.

## Data Availability

The original contributions presented in the study are included in the article/Supplementary material, further inquiries can be directed to the corresponding author.
